# Novel and Accessible Physical Recycling for Expanded Polystyrene Waste with the Use of Acetone as a Solvent and Additive Manufacturing (Direct Ink-Write 3D Printing)

**DOI:** 10.3390/polym15193888

**Published:** 2023-09-26

**Authors:** Rubén García-Sobrino, Alejandro Cortés, Rocío Calderón-Villajos, Jorge G. Díaz, Marta Muñoz

**Affiliations:** 1 Department of Applied Mathematics, Materials Science and Engineering and Electronic Technology, Universidad Rey Juan Carlos, Calle Tulipán s/n, 28933 Móstoles, Spain; ruben.sobrino@urjc.es (R.G.-S.);; 2 School of Mechanical Engineering, Industrial University of Santander, Bucaramanga 680002, Colombia; jgdiazro@correo.uis.edu.co

**Keywords:** circular economy, plastic waste recycling, expanded polystyrene, additive manufacturing, Direct Ink Write

## Abstract

The current high production of plastics has prompted the exploration of alternative pathways to facilitate recycling, aiming for a progressively sustainable society. This paper presents an alternative and affordable technology for treating waste expanded polystyrene (EPS) mixed with acetone in a 100:1 volume ratio to be used as 3D printing ink for Direct Ink Write technology. In order to optimize the printing parameters, a comprehensive study was conducted, evaluating different needle diameters, printing speeds, and bed temperature values to achieve homogenous pieces and a highly repeatable 3D printing process. Results showed that the main optimum printing parameters were using needles with diameters of 14 to 16 G and printing speeds ranging from 2 to 12 mm/s, which were found to yield the most uniform ribbons. Increasing the bed temperature, despite favoring acetone evaporation, led to the generation of more heterogeneous structures due to void growth inside the printed ribbons. Thus, employing room temperature for the bed proved to be the optimal value. Lastly, a comparative study between the starting material and the EPS after the printing process was conducted using FTIR-ATR and GPC analyses, ensuring the preservation of the original polymer’s integrity during physical recycling.

## 1. Introduction

During the 20th century, the synthesis and production of new plastics have greatly contributed to the achievements and advances developed by mankind to date. In fact, they have become so important that they now accompany most human beings in their daily actions. All this has led to the fact that since the 1950s, the production of plastics has been increasing by 10% each year, reaching an estimated annual production of 380 million tons [1,2]. As already mentioned, the presence of plastics in almost every daily human activity seems to respond to the trend that this high production value will continue to increase in the coming decades [3]. The direct negative consequence of this fact is the problem generated by plastic waste after the end of its useful life, with disastrous environmental, health, and economic consequences [1,4]. As an alternative strategy and with the main objective of an increasingly sustainable society, the circular economy (CE) emerges [5]. This idea, highlighted within the European ecological transition process (European Green Pact, EC, 2019 [6]), is currently the subject of a large number of articles in the literature with the aim of finding alternative methods to the problem generated by the enormous impact that plastic waste generates [7,8,9].

Within plastics, materials such as polyethylene (PE), polystyrene (PS), polypropylene (PP), polyvinyl chloride (PVC), or polyethylene terephthalate (PET), all belonging to the thermoplastic family, represent around 80% of the world production of all polymers [10]. In the case of PS, its hydrophobic nature and high molecular weight make it difficult to degrade, unlike other hydrocarbon chains [11]. Within the framework of this work, expanded polystyrene (EPS), also known as Styrofoam, is one of the most widely used modifications of PS at present. It is characterized by having a high amount of air inside (about 92–98 wt.%), which makes them ideal as containers that maintain the temperature of refrigerators or food products due to their low density and high isolating capacity [11,12]. One important concern with this waste material is its high specific volume, which complicates different storage processes. In this context, there are many proposals for the recycling of this type of polymer to avoid accumulation in landfills, categorized into three levels: advanced recycling based on chemical reactions such as pyrolysis or gasification; physical recycling involving physical processes such as defoaming and dissolution; and mechanical recycling such as grinding or extrusion [13]. Some advanced techniques found in the literature on EPS recycling include the study by Ming Yang et al. [14], in which expanded polystyrene is recycled through a new formulation for binder, producing a high-performance adhesive; on the other hand, the research of Ali N. Siyal et al. [12], in which EPS is chemically recycled to obtain a new functional polymer, phenyl thio-semicarbazone, for the treatment of lead-contaminated water; also, by Ruochen et al. [15], a photocatalytic method is proposed to oxidize PS to aromatic oxygenates. This research work focuses on physical recycling through a defoaming process using acetone as a solvent to remove the air molecules occluded in the expanded polystyrene residue and obtain a polystyrene paste used as an ink for 3D printing [13].

Additive manufacturing (AM) technologies have been a breakthrough since they were first positioned owing to their key advances, such as the ability to create complex structures in one single piece without assemblies, mass customization, or the manufacturing of unique parts without significantly increasing the manufacturing costs, and material waste minimization, among others [16,17]. Concerning the study of this work, the use of recycled polymers in additive manufacturing has become an interesting topic in the scientific community during the last few years due to the environmental concerns mentioned above [18]. There are several research studies using recycled thermoplastics for additive manufacturing, as summarized by Mikula et al. [19] and Nguyen et al. [18]. In this regard, the most studied ones are polylactic acid (PLA), acrylonitrile butadiene styrene (ABS), polyethylene terephthalate (PET), high-density polyethylene (HDPE), polypropylene (PP), polystyrene (PS), and high-impact polystyrene (HIPS). Nevertheless, most effort has been put into obtaining filaments for Fused Filament Fabrication (FFF) 3D printing technology, which consists of extruding the filament through a heating nozzle and selectively depositing the thermoplastic material layer by layer until the 3D geometry is completed [20]. In this regard, Yoshimura et al. [21] used different ratios of acetone and ethanol to dissolve several mixes of polymers such as poly methyl methacrylate (PMMA), PLA, ethyl methacrylate (EMA), butyl methacrylate (BMA), and methyl methacrylate (MMA), among others. The copolymers were used to fabricate wires to be subsequently used in a FFF printer. On the other hand, some interesting research works have been found using EPS for FFF, such as the one of Turku et al. [22], who performed a chemical, thermal, and mechanical characterization of the 3D printed filaments; the one of Bartolomei et al. [23], who used EPS with a biodegradable solvent to manufacture 3D printed prototypes and finishing construction materials; and the research works of Chu et al. [24] and William et al. [25], who investigated with recycled polystyrene and polypropylene blends. However, all these studies produced filaments that needed to be melted subsequently by carrying out an extrusion process, which can be quite energy-consuming on an industrial scale.

Moreover, Direct Ink Write (DIW) 3D printing allows to shorten the manufacturing process and reduce energy consumption since it is a suitable technology that consists of extruding a viscous paste through a pressurized nozzle [26]. The DIW technique has recently been used for soft metal alloys [27], ceramics [26], and polymers [28], producing filaments to be later used in FFF. In this context, it allows one to extrude, at room temperature, a paste based on thermoplastic material diluted in some solvent without using complex and energy-consuming processes of extrusion and winding to obtain the feedstock for the 3D printer. As far as the authors know, this is the first research study using EPS-acetone paste to obtain 3D-printed parts using the DIW technology.

In the present manuscript, a simple, alternative, and accessible strategy for recycling EPS waste has been studied that can be carried out by any type of user whenever a DIW printer is available. Briefly, this process develops three simple steps that can be enumerated: the collection of the EPS waste, the defoaming of the waste in acetone (C_3_H_6_O), and, finally, the treatment of the mixture with the 3D printing technology, more specifically, with the Direct Ink Write 3D printing technology described previously. To develop this last step, a simple ink formulation made up of the EPS-acetone mixture (EPS-ink) generates a paste of high plasticity and viscosity that allows extrusion with the 3D printing equipment. Once the EPS-ink has been evaluated and characterized, the manuscript describes, on the one hand, the optimization of the 3D printing process to obtain homogeneous and repeatable prints and, on the other hand, a detailed study to demonstrate the null degradation of the original material by this physical recycling alternative treatment [13].

## 2. Materials and Methods

### 2.1. Materials

EPS waste was collected from the research centers associated with this manuscript: Universidad Rey Juan Carlos (Spain) and Universidad Industrial de Santander (Colombia). The starting EPS used for the study came from different sectors, such as the packaging of different electronic elements or food packaging for food deliveries. In all cases, it was carefully cleaned and dried before any further processing. On the other hand, acetone solvent (99.6%) GLR from Labkem has been used to defoam and minimize the volume of the recycled EPS collected.

### 2.2. Preparation and Evaluation of the EPS-Ink

An approximate mixture of EPS and acetone in a volume ratio of 100:1 was used to evaluate the possibility of using EPS waste to obtain an alternative and affordable recycling process from Direct Ink Writing 3D printing technology. It is necessary to emphasize that, due to the fast evaporation of the acetone and to maintain the homogeneous characteristics of the EPS-ink, a superficial excess of acetone is added, which is removed prior to the corresponding study.

Viscosity characterization of the EPS-ink was carried out at room temperature (25 °C) using a Brookfield DV2T viscometer and associated Rheocalc T software (v1.2.19). A cylindrical spindle N-LV3 (63) [29] was used to introduce into the solution to apply shear stress to provide the viscosity value for the mixture in which it is immersed. Three viscosity measurements were performed for reproducibility. The viscosity was measured at different revolutions per minute (RPM): from 0.1 to 0.7 RPM, and repeated three times for error analysis.

### 2.3. Manufacturing by 3D Printing and Optimization of the Process

**Figure 1:**
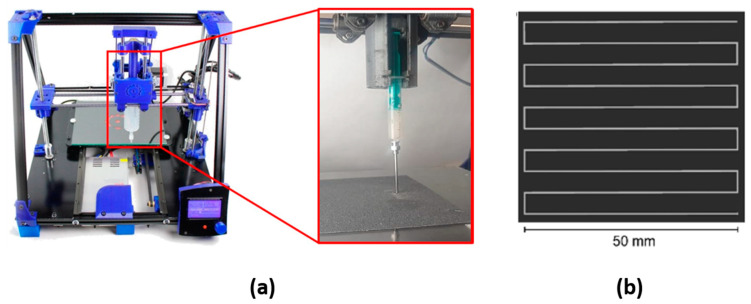
(**a**) BCN3D Plus printer: image with the additional module Paste Extruder, which allows the printing of fluids from commercial disposable syringes. (**b**) Design of the part to be printed by CATIA v5 software (plan view).

The DIW 3D printing equipment used in the present study is a BCN3D Plus printer (Barcelona, Spain) with an additional module called Paste Extruder. The latter element was modified to allow printing from disposable syringes, facilitating the accessibility of the technology presented to different user levels (see Figure 1a). This added element was also printed with FFF technology using a BQ Witbox printer.

The previously described EPS-acetone ink (EPS-ink) was used as printing ink. It is important to note that, in order to improve the homogeneity of the printing process, the acetone supernatant belonging to the ink was removed before loading the syringes.

On the other hand, to improve the adhesion of the EPS-ink solution to the platform, the printing bed was coated with SiC-P120 sandpaper (R_a_ = 24.05 ± 1.27 µm) purchased from QATM Quality Assured.

Finally, the 3D model used to optimize the printing parameters was designed using the CATIA v5 software (Dassault Systèmes, Velizy-Villacoublay, France). It is composed of a circuit of 10 continuous parallel lines connected to each other to avoid interruption of the printing flow (Figure 1b).

In order to optimize the printing process, some parameters were varied to study their effect in terms of homogeneity and reproducibility of the printing process: diameter of the needle, printing speed, and finally, temperature. On the other hand, some printing parameters, controlled using the Slic3r 1.3.0 software companion, were fixed. Here, the extrusion multiplier was kept at a value of 2 units, the nozzle diameter was set to 2 mm, and the retraction was set to 0.5 mm.

Concerning the optimization process, depending on the needle diameter, different diameter stainless steel blunt tip needles were purchased from BENECREAT with a length of 50 mm. The diameters purchased were 10, 12, 14, 16, 18, 20, 22, and 24 gauges (G), equivalent to 3.40, 2.77, 2.11, 1.65, 1.28, 0.91, 0.72, and 0.57 mm, respectively.

A gravimetric analysis was carried out as a complementary study to the printing process as a function of needle diameter. For the proposed kinetics, one drop of ink for each needle diameter at room temperature (25 °C) was analyzed. The percentage weight loss of acetone (*W_L_* (%)) was defined as the percentage of mass lost per unit of time by solvent evaporation. This value was calculated from Equation (1).

(1) $$W_{L}\;\left(\%\right)=100-\left(\frac{Wt}{Wi}\right)\times 100$$

where *W_i_* is the initial mass value of the droplet and *W_t_* is the mass value of the droplet analyzed at each proposed time. The study was analyzed in triplicate.

In addition, a control study was also conducted to discuss the need for using a needle with the syringe during printing and the need for using silicone oil as a lubricant to reduce friction forces. Here, the needle tips were dip-coated in silicone oil prior to the extrusion process to reduce friction between the ink and the needle. See Figure S1a for more information about the aforementioned studies.

In the case of the printing speed optimization process, different speeds were proposed: 2, 8, 12, 16, 25, and 30 mm/s. In order to reduce the number of tests, the effect of the printing speed on the ribbon’s homogeneity was carried out just with the optimum needle diameter obtained from the previous study. Finally, heating bed temperature possibilities (25, 40, 45, 50, and 55 °C) were studied. Prior to this study, isothermal thermogravimetric analysis (TGA) was performed on TGA SDT650 equipment from TA Instruments to measure the vaporization speed as a function of the temperature. The tests were carried out at controlled and constant temperatures of 40, 45, 50, and 55 °C in an air atmosphere of 100 mL/min. The study corresponds to a constant temperature value for 60 min to observe the weight loss of the samples. Measurements were evaluated in triplicate.

The printing quality, in terms of homogeneity and reproducibility of the 3D printing process, was studied by image analysis using the ImageJ software (1.54f, National Institute of Health, Bethesda, MD, USA). Here, the ribbon’s width was measured from pictures taken in plain view. Several measurements for each specimen were carried out in order to obtain a robust study. The ribbon’s width deviation was obtained as the percentage of the standard deviation with regard to the width of the printed ribbons. In this context, a higher ribbon width deviation suggests higher heterogeneity during the extrusion process and, thus, lower repeatability of the 3D printing process. On the other hand, the cross-section images were obtained from the printed ribbons. For this case, slices of each sample were embedded in resin and prepared according to the AkaSel kit’s instructions (a mix of Aka-Resin and Aka-cure elements). Pictures were taken using a Leica/S6D stereo microscope equipped with a Leica DFC320 camera (Wetzlar, Germany) associated with the Leica LAS software (1.4.5).

### 2.4. Comparative Analysis of 3D-Printed Material vs. Original Material

To control the chemical composition of the materials after the printing process, Fourier-Transform Infrared (FTIR) characterization was carried out with a Nicolet™ iS50-ISASA spectrophotometer with Omnic software (v9.13, Waltham, MA, USA), in the 4000–400 cm^−1^ range and with a resolution of 4 cm^−1^ using Attenuated Total Reflectance (ATR) mode.

In addition, weight-average and number-average molecular weights (Mw and Mn, respectively) and polydispersity index (PDI) were measured by gel-permeation chromatography (GPC) with the use of three columns of PL Gel Olexix GPC. The system was calibrated with a narrow molecular weight distribution of standard polystyrene.

Finally, differential scanning calorimeter (DSC) measurements were made on a heat flow DSC equipment from Mettler (Greifensee, Switzerland) with a heating and cooling rate of 10 °C/min and a nitrogen flow of 50 mL/min. The sample was heated from 30 to 200 °C in a nitrogen environment. The glass transition temperature (Tg) was calculated using the turning point analysis method.

## 3. Results

### 3.1. EPS as Waste Diluted in Acetone: Evaluation and Preparation of the Ink for 3D Printing

As mentioned above, this manuscript aims to present an alternative and accessible method for the physical recycling of EPS waste using acetone as a solvent with the support of Direct Ink Write 3D printing technology.

**Figure 2:**
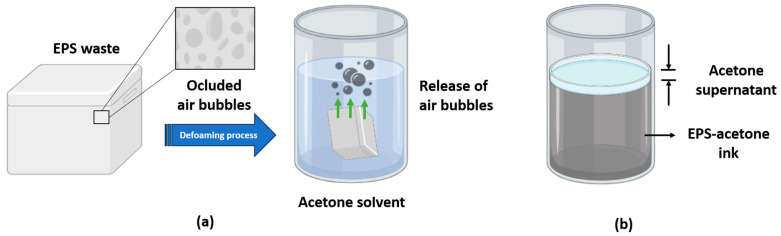
Basic scheme of: (**a**) the process of releasing occluded air (the defoaming process) from the EPS residue in the presence of acetone; and (**b**) two-phase formation after EPS saturation in acetone.

Based on the experiment and schematized in Figure 2a, the immersion of the EPS waste into acetone induces a defoaming process, which also causes a change in their physical characteristics from a solid elastic behavior to an increased plasticity. In this context, the aforementioned mixture can be used to obtain parts by using the DIW 3D printing technology, as this EPS-ink is able to flow when pressure is applied to it. Moreover, the defoaming process, thanks to the high amount of air contained in the material, occurs in a few seconds when the EPS is in the presence of the solvent, releasing the occluded air bubbles, as observed in the attached Supplementary Video S1. In the case of this work, and thanks to the high specific volume value of EPS, a mixture with a volume ratio of approximately 100:1 EPS-acetone was obtained (see the scheme in Figure 2b and a real picture in the Supplementary Material, Figure S2, where the EPS-acetone mixture with saturation of the latter is shown, with two clearly differentiated phases showing the maximum possible dilution volume carried out).

**Figure 3:**
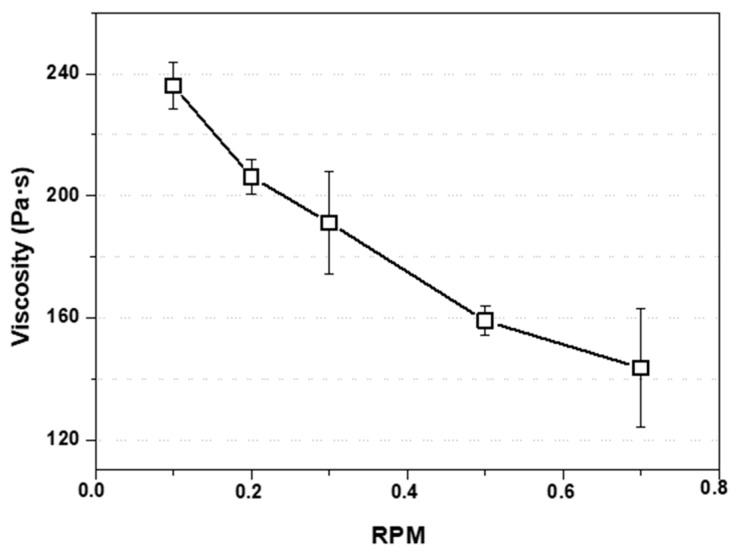
Viscosity values of the EPS-acetone mixture to be printed. Measurements from 0.1 to 0.7 RPM.

For the viscosity test shown in Figure 3, and as mentioned in Materials and Methods, acetone supernatant was removed prior to the test. In the figure shown, viscosity was plotted as a function of RPM at 25 °C, where it can be seen that the viscosity value decreases as the shear rate increases. This behavior, characteristic of non-Newtonian pseudoplastic fluids, generates in the macrochain a situation known as shear thinning, which implies that the macromolecule temporarily aligns with the spindle wall during the stress, generating a decrease in the viscosity value as the test speed increases [29].

### 3.2. 3D Printing Parameter Optimization

**Figure 4:**
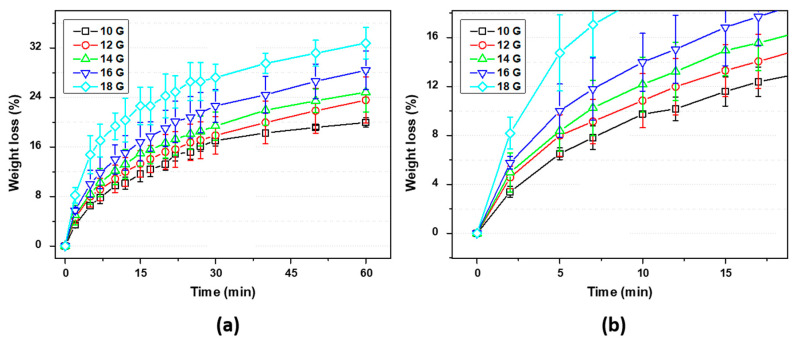
Kinetic gravimetry test: (**a**) Needles with different diameters were evaluated (10, 12, 14, 16, and 18 G). (**b**) On the right, a magnification plot is presented to analyze the first stages of the situation.

Prior to the optimization of the needle diameter, a gravimetric analysis was carried out with each of the needles: 10, 12, 14, 16, 18, 20, and 24 G (see Figure 4). In this test, the mass loss due to the evaporation process of a drop of EPS-ink was monitored at room temperature (25 °C) for 60 min. It is observed that most of the samples presented a typical solvent evaporation curve. However, the force needed to extrude the EPS-ink through the needle when using the 20 and 24 G ones was too high to be carried out manually, so these needles were discarded for this study. Again, a clear trend is observable in which the needles of larger sections lose less mass compared to the samples of greater diameter during the 60-min test. This difference is accentuated in the early stages of the test (see magnification in Figure 4b), indicating the rapid evaporation of the mixture due to the volatility of the solvent. Given the fairness in terms of chemical composition of the ink, the importance of the amount expelled after the extrusion process is observed, where the samples with a lower amount of EPS-ink lose a higher mass percentage, demonstrating the importance of the geometry of the specimen in kinetic studies by gravimetry, as other authors have shown [30]. Here, the evaporation process begins on the surface of the printed ribbons, generating a thin layer that hinders the evaporation process of the remaining acetone. Thus, the higher the volume of the deposited droplet, the lower the evaporation rate. Finally, it is necessary to emphasize that samples 10, 12, 14, and 16 G have shown similar behavior where there are no differences in terms of error between them, so a priori, they show a similar behavior pattern as they have a similar diameter.

**Figure 5:**
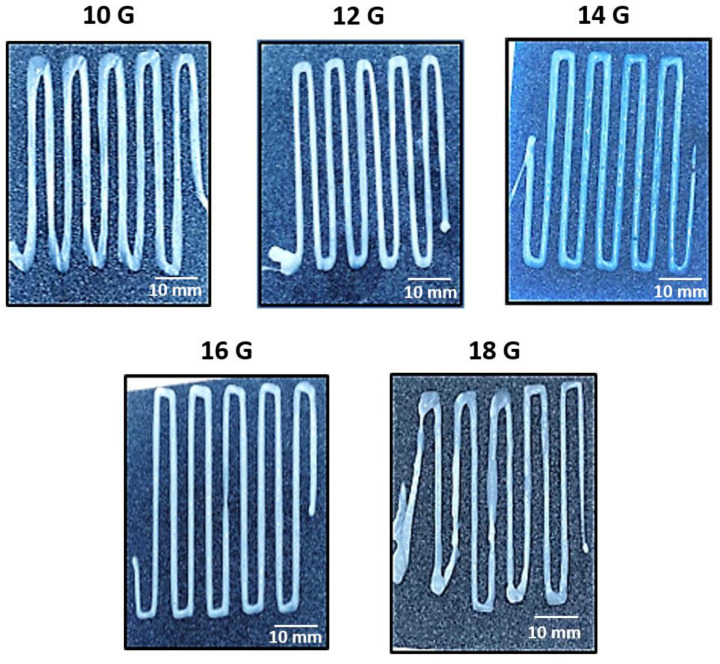
Plan views of the different 3D printed specimens as a function of the needle diameter (10, 12, 14, 16, and 18 G). The printing speed was kept constant at 2 mm/s for all studied conditions.

Once the preliminary study took place, the optimization of the needle diameter was carried out with the 3D printer at a constant printing speed of 2 mm/s. The selected design (previously described by A. Cortés et al. [31] in his manuscript on a CNT-Doped Nanocomposites printing optimization process and shown in Figure 1b) forces the ink to form linear patterns with turns of 90° during the extrusion process, in which the ability to print simple designs with changes of direction is studied as a preliminary test to achieve in the future the 3D printing of more complex geometry designs. In Figure 5, it is shown that it was possible to print specimens only using needle diameters of 10, 12, 14, 16, and 18 G, as in the preliminary kinetic gravimetry test. This is due to the fact that the 3D printing equipment is not able to apply enough force to extrude the EPS-ink through the needles with lower diameters (20 and 24 G). In this sense, the ribbons obtained with the 18 G needle showed the worst homogeneity since the pressure needed to extrude the EPS-ink is within the limit of the equipment, causing a slip among the gears of the extruding module of the 3D printer. This qualitative analysis can be supported by the results of the ribbon’s width deviation, shown in Table 1. Here, ribbon’s width deviation obtained with the 18 G needle is the highest of all the tested conditions due to the reasons mentioned above. Taking this into account, the 18 G needle was discarded for the optimization of the 3D printing process. As the value of G is reduced (and, as it was mentioned, diameter increases), the specimens show more homogeneous structures, even when performing sudden 90° turns. Nevertheless, the sharpness of the printed ribbons in the 90° turns significantly decreases when using the thickest diameters, which may be attributed to a higher force pulling the deposited ribbons carried out by the needle during printing. Again, these results agree with the results of the ribbon’s width deviation shown in Table 1.

Therefore, the nozzle diameters that showed the best results in terms of homogeneity of the ribbons and reproducibility of the printing process are 14 and 16 G.

The control study, where the need for using a syringe when printing was analyzed (Figure S1a), suggests that the needle length of 50 mm may stabilize the ink flow through it, leading to a considerably higher homogeneity and repeatability of the printed parts.

**Figure 6:**
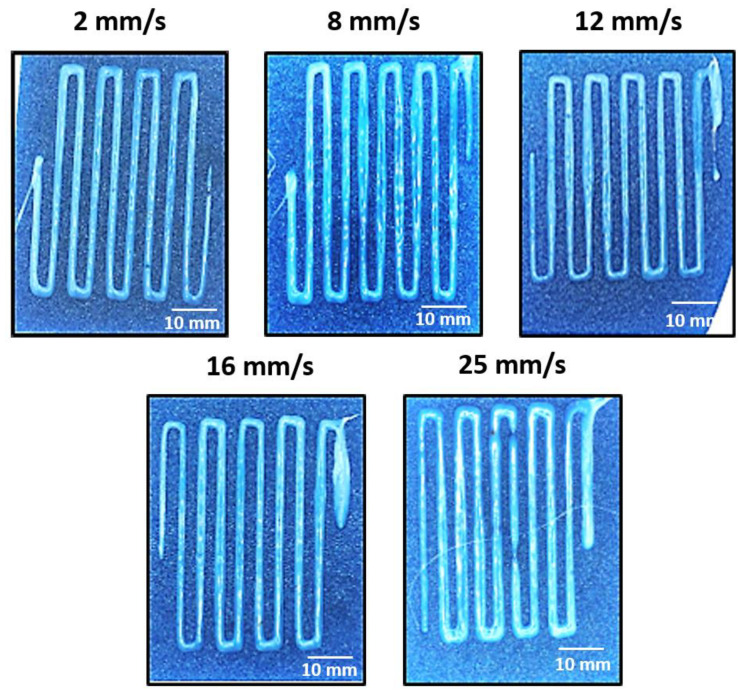
Plan views of the different 3D printed specimens as a function of the printing speed (2, 8, 12, 16, and 25 mm/s). The needle diameter was kept constant at 14 G for all studied conditions.

Regarding the printing speed optimization, although the 14 and 16 G needle diameters showed the best results in terms of homogeneity and repeatability of the 3D printing process, the 14 G needle diameter was selected and used for this study in order to reduce the number of tests. Here, different speeds ranging from 2 to 25 mm/s were proposed. Plan views of the printed specimens are shown in Figure 6, and the results of the analysis of the ribbon’s width are summarized in Table 2. Here, the homogeneity of the printed ribbons remains almost constant in a speed range from 2 to 12 mm/s. Nevertheless, the increase in the printing speed at higher values induces an increase in the ribbon’s width deviation, which may be attributed to the higher pulling forces applied to the deposited ribbon by the needle when moving, leading to stick-slip mechanisms between the deposited ribbons and the substrate and, thus, to a lower repeatability of the 3D printing process. Therefore, from these observations and for the selected 3D printer, a printing speed ranging from 2 to 12 mm/s is suggested as optimum 3D printing conditions.

**Figure 7:**
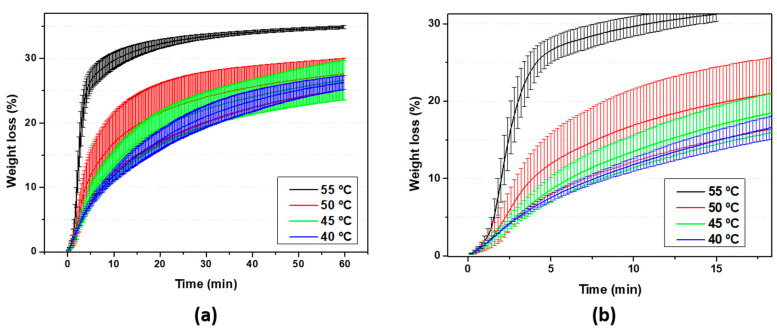
(**a**) Weight loss of acetone for EPS at different temperatures and (**b**) magnification of the ramp of the curve.

Finally, the bed temperature used for the printing process was optimized. The initial purpose of increasing the bed temperature during the 3D printing stage was to favor the evaporation of acetone in the shortest possible time. In Figure 7, thermogravimetric analysis at different isotherms (40, 45, 50, and 55 °C) was represented for 60 min. Here, it can be seen how the increase in temperature induces a faster (see magnification in Figure 7b) and greater weight loss within 60 min of the test, as expected. It is seen in Figure 7 how the largest weight loss is obtained at 55 °C compared with the other measured temperatures of 40, 45, and 50 °C. This might be explained by the fact that the acetone vapor pressure value at 55 °C (0.94 atm) [32] is almost equal to atmospheric pressure and the vaporization enthalpy value is 29,488.6 J/mol [33], which causes the boiling point to be reached and the release of gaseous acetone molecules to substantially accelerate.

**Figure 8:**
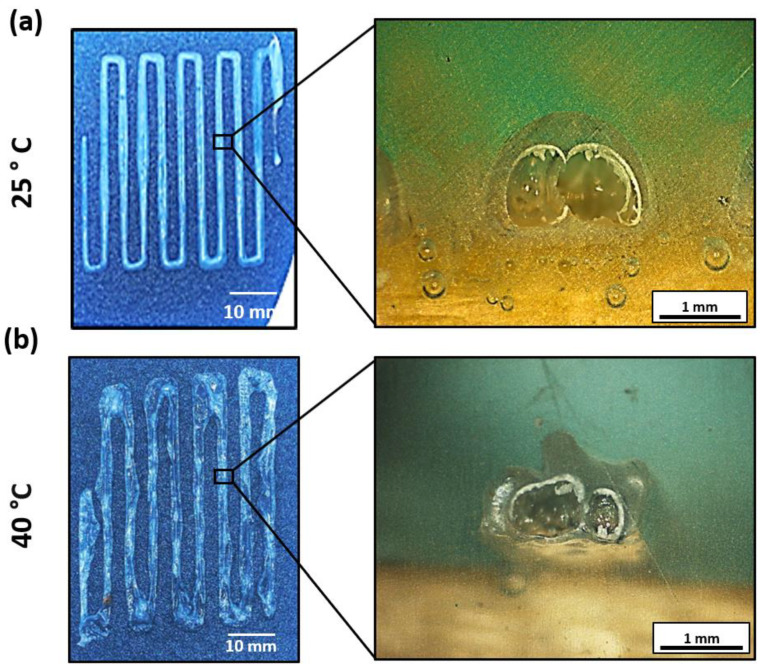
Plan and cross-sectional views of the specimens printed using the 14 G needle, a printing speed of 12 mm/s, and a bed temperature of (**a**) 25 °C and (**b**) 40 °C, respectively.

To transfer this idea to the 3D printing process, a study was proposed with a bed temperature value of 40 °C. Figure 8 shows a comparison of two specimens printed with the same needle diameter (14 G) and printing speed (12 mm/s) but with different bed temperatures (25 and 40 °C, respectively). Here, a higher heterogeneity can be observed for the specimen printed with the highest temperature, which is also in accordance with the quantitative study of the ribbon’s width deviation shown in Table 3. This behavior can be attributed to the formation of the thin, dry layer previously mentioned in the kinetic gravimetry test. In this regard, this layer hinders the release of the remaining solvent by evaporation, acting as a hardened barrier and, thus, leading to void growth and, therefore, distortion of the printed ribbons. To support these findings, the cross-sectional pictures shown in Figure 8a,b, evince that the ribbons printed at 40 °C present a lobed cross-sectional profile due to the void growth instead of the conventional semicircular section of the ribbons printed at room temperature. In this context, the trapped gas (air and acetone) within the EPS is trying to expand due to the heated printing bed. Therefore, the thin, dry membrane containing the bubble experiences normal stress, deforming the most at the thinnest region. The ribbon may warp if the trapped gas forms a non-symmetric membrane, as shown in Figure 8b. Thus, the increase in the 3D printer bed temperature to enhance the evaporation of the remaining solvent was discarded.

In summary, from the observed experiments, the optimal printing conditions are using a needle diameter ranging from 14 to 16 G, a printing speed from 2 to 12 mm/s, and a bed temperature of 25 °C. Moreover, the use of a large needle, as well as silicone oil, is highly recommended (see optimal 3D printing conditions in a video in Supplementary Video S2). 

**Figure 9:**
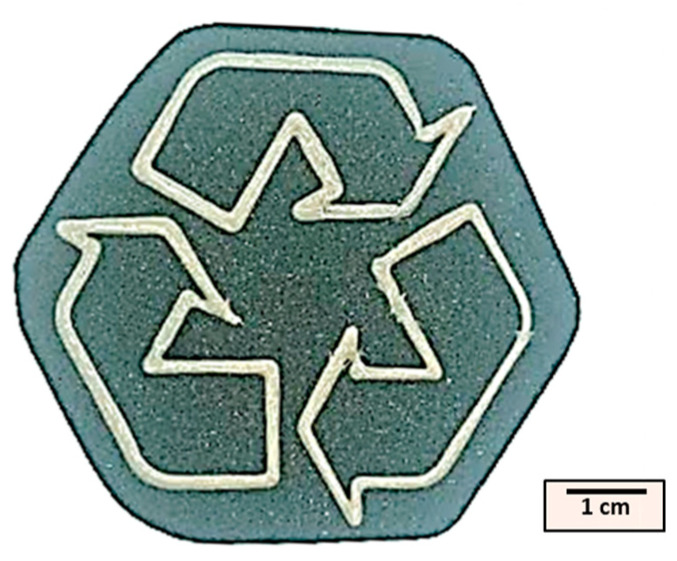
Proof-of-concept of a recycling symbol obtained using the optimum printing parameters (14 G, 12 mm/s, and 25 °C).

Finally, Figure 9 shows a proof-of-concept carried out with the optimum printing parameters. Here, the ability to print geometries with different angles and curvatures has been demonstrated.

### 3.3. Comparative Analysis of Printed Material vs. Original Material

Once the printing process was optimized, obtaining a wide range of use speeds for two of the proposed needle diameters (14 and 16 G), the demonstration of the null compositional damage of the sample after the alternative process of mechanical recycling proposed in this work was exposed next. Defining physical recycling as a process of a physical nature that does not modify the macrochain of the polymer for its recirculation [13], different comparative studies are presented in the present point for its demonstration. To facilitate the compression of the same, EPS−1 will define the virgin EPS sample, that is, prior to the defoaming process in acetone. On the other hand, EPS−2 refers to the material during the acetone defoaming process, and finally, EPS−3 defines the final filament once printed.

**Figure 10:**
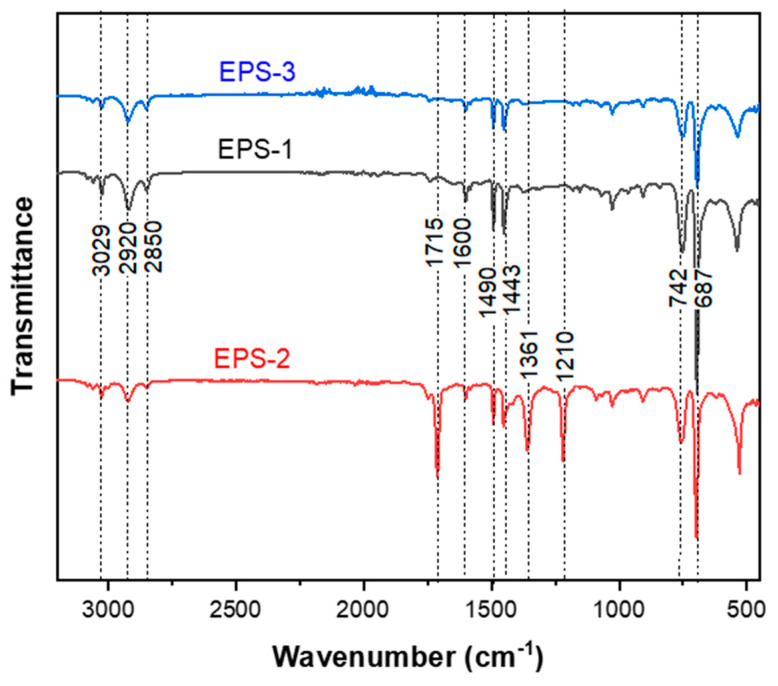
Fourier transform infrared (FTIR) spectroscopy of the samples: EPS−1 (EPS waste in black color), EPS−2 (EPS defoamed in acetone in red color), and EPS−3 (final filament once printed in blue color).

The FTIR spectra of EPS−1, EPS−2, and EPS−3 are shown in Figure 10. It can be seen that the three materials correspond to the same polymer and that there is no evidence of degradation since there are no bands corresponding to oxygenated groups. Characteristic bands of EPS are observed in the three curves in the region from 3150 to 2700 cm^−1^. The band at 3029 cm^−1^ is given by the stretching vibrations of the C-H bond of the aromatic ring, and the bands at 2920 and 2850 cm^−1^ correspond to the stretching of the CH-(CH_2_) bond of the alkyl part of the PS. The characteristic bands of substituted aromatic rings observed in the region from 2000 to 1670 cm^−1^, where stretching vibration bands of the C-C bond of aromatic compounds are presented in pairs at 1600 and 1582 cm^−1^ and 1490 and 1443 cm^−1^. In addition, the mono-substitution region is observed at 742 cm^−1^, corresponding to the out-of-plane bending vibrations of five adjacent hydrogen atoms in the aromatic ring, and at 687 cm^−1^, corresponding to the out-of-plane bending vibrations of the aromatic ring [34]. To see the acetone saturation (EPS−2), the most characteristic band of the acetone is the stretching vibration of the carbonyl group at 1715 cm^−1^, where it is clearly observed that EPS−1 and EPS−3 do not have this band typical of ketone groups, and neither do the CH bending bands at 1210 and 1361 cm^−1^, which assures the non-presence of the same after the printing process is carried out. Unfortunately, other bands coincide with some bands of the PS functional groups, such as the symmetric and asymmetric stretching and bending vibration bands of the methyl groups of the acetone at 2800–3000 cm^−1^ and 1350–1450 cm^−1^.

**Figure 11:**
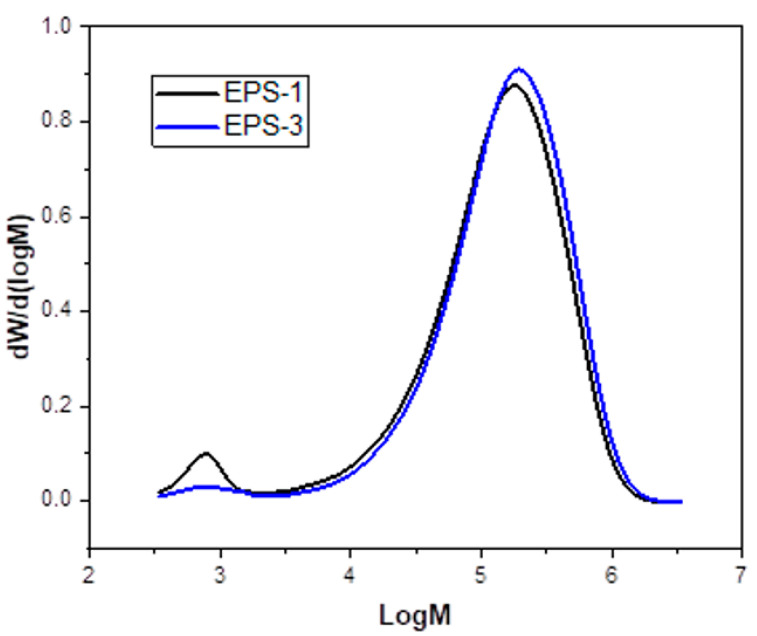
MWD of the EPS−1 (EPS waste in black color) and EPS−3 (final filament once printed in blue color) samples used in this study.

An analysis of the molecular weight distribution (GPC analysis) after the DIW printing process is represented in Figure 11. The MWD of samples EPS−1 and EPS−3 is shown. In contrast, the numerical values of the mass average (Mw) and number average (Mn) molecular masses with the corresponding polydispersity index (PDI) of the samples are provided in Table 4. As observed, there is no notable change in the mass-averaged and number-averaged molecular weights in the two samples, EPS−1 and EPS−3, respectively. The small variations observed can be associated with the fact that the EPS residues come from different sources and manufacturing processes, so consequently, properties such as Mw and Mn may show small variations. In addition, all the samples display single modal peaks with narrow MWD, which is similar to the observations made by Cella et al. [35], Caceres et al. [36], and Zhang et al. [37]. This observation is accompanied by close PDI values (3.26 and 3.07, respectively; see Table 4). The steps of defoaming and 3D printing do not significantly affect the average molecular weights obtained. It can be seen how EPS−1 has a low molecular weight component corresponding to the additives that are added to improve the properties of EPS for different industrial applications. For the EPS−3 curve, this peak has practically disappeared due to the fact that in the process of defoaming the polymer in acetone, part of these soluble additives in the solvent are eliminated. In conclusion, there is no oxidative degradation of EPS during the printing process.

In order to know the influence of the proposed process on the thermal transition values, calorimetric studies are included in Table 4. In it, it can be observed how the results of EPS−1 and EPS−3 in terms of glass transition temperature are similar for both cases (100.0 and 104.5 °C, respectively). As previously mentioned, the original mixture of starting materials can generate heterogeneities in the comparative measurement, as observed in the GPC graph (see Figure 11). Thus, the studies shown indicate that the process presented in this manuscript does not generate modifications in terms of temperature transition values.

In conclusion, and based on the analysis, the defoaming and printing process with DIW technology does not cause damage to the polymeric chain as no chemical reaction is involved, thus ensuring recycling of a physical nature [13]. As already mentioned, the development of new recycling methodologies favors the proper management of end-of-life polymer waste. Yang W. et al. [38] recognize the different ways of thermoplastic recycling currently existing and point out in particular the importance of the development of new ways of physical nature. In this case, the present work shows a simple, accessible, and efficient technology with no highly specialized equipment, as opposed to chemical methods requirements [38]. In addition, in the case of extrapolating this work to a semi-industrial level, the process is fully scalable. In fact, any company that works with packaging or EPS waste and possesses a conventional 3D printing machine by FFF can freely download files to cheaply assemble a DIW device that allows the proposed recycling process.

It should be noted that despite the ease of assembly and preparation technology described above, the use of acetone as a solvent requires a series of precautions to avoid the development of associated hazards. On the one hand, in healthy terms, acetone is a liquid that, in continuous exposure, can cause dry skin; likewise, inhalation of vapors can cause drowsiness and dizziness, so the use of this technology is highly recommended in rooms with access to ventilation [39,40]. On the other hand, many authors have shown their concern about the study of flammable solvents such as acetone, the protagonist of this work; therefore, correct and adequate use is essential to ensure good practice, including, if possible, even inert gases added to limit associated danger (CO_2_, He, N_2_, or Ar) [41,42]. However, in this research, the amount of acetone used was minimal compared to the amount of recycled polystyrene. Only a solvent supernatant is required to avoid the loss of plasticity of the polymer, as shown in Figure 2b.

## 4. Conclusions

A new alternative, simple, and accessible route for the physical recycling of EPS waste using DIW 3D printing technology has been presented in this research work. A mixture of EPS waste and acetone in a volume ratio of 100:1 has been used as printing ink.

First, an optimization of the 3D printing process was carried out to obtain homogeneous parts and high repeatability. The results of the optimization study suggest using needles ranging from 14 to 16 G (2.11 and 1.65 mm, respectively) in diameter. Here, an excessive needle diameter would lead to distortion in the printed ribbons caused by the pulling forces induced by the syringe on the ribbons during printing, especially when performing turns of 90°. In contrast, the lowest diameters could lead to slippage of the paste extruder module gears due to the high forces applied by the 3D printer. Moreover, a printing speed of 2 to 16 mm/s is suggested since higher speeds could induce stick-slip mechanisms between the deposited ribbons and the substrate. Finally, it is suggested to print without increasing the bed temperature above room temperature since it enhances the void wrought inside the printed ribbons, leading to distortion in their cross-sectional area. Additionally, control tests suggest that using 50-mm-long needles stabilizes the ink flow. Moreover, dip-coating the needles with silicone oil before printing reduced the friction forces between the ink and the needle when printing. The proof-of-concept, carried out with the optimum printing parameters, proved the suitability of the technique to print parts with different angles or curvatures.

Furthermore, in order to demonstrate that the presented process does not damage the initial polymeric macrochain, different studies have been carried out in comparison with the original material. On the one hand, the surface analysis by FTIR-ATR has shown that the initial and final samples maintain the characteristic peaks of EPS waste; in the same way, the studies carried out on the basis of the molecular weight have shown no modification of the recycling process on the macro chain; finally, the materials did not show any variation in terms of thermal transition values either.

In summary, these results indicate the feasibility of this physical recycling technology for EPS waste, which is necessary at present due to the increasing production of this material and its complicated storage due to its high specific volume. In addition, the ease and accessibility of the process presented an added value since any user who wants to recycle EPS waste can do so by using acetone as a solvent and a 3D printer that allows extrusion with the use of disposable syringes.

## Figures and Tables

**Table 1:** Ribbon’ width deviation as a function of the needle diameter (speed printing was 2 mm/s).

Needle Diameter	10 G	12 G	14 G	16 G	18 G
Average width (mm)	2.30 ± 0.26	1.94 ± 0.18	1.83 ± 0.17	1.84 ± 0.16	1.65 ± 0.35
Ribbons width deviation (%)	11.49	9.21	9.42	8.44	21.31

**Table 2:** Ribbon’ width deviation as a function of the printing speed (diameter of the needle was 14 G).

Printing Speed (mm/s)	2	8	12	16	25
Average width (mm)	1.84 ± 0.22	2.15 ± 0.24	1.93 ± 0.22	2.04 ± 0.37	3.06 ± 0.47
Ribbons width deviation (%)	11.73	11.06	11.26	18.38	15.24

**Table 3:** Ribbon’ width deviation as a function of the bed temperature (diameter of the needle was kept at 14 G, and speed printing was 12 mm/s).

Bed Temperature (°C)	25	40
Average width (mm)	1.93 ± 0.22	2.46 ± 0.63
Ribbons width deviation (%)	11.26	25.73

**Table 4:** Information on plastic characteristics extracted from GPC results and calorimetry analysis.

Sample/Label	EPS−1	EPS−3
Mn (g/mol) × 10^4^	6.55	7.71
Mw (g/mol) × 10^4^	21.32	23.71
PDI	3.26	3.07
Tg (°C)	100.0	104.5

## Data Availability

Data is available upon request.

## Supplementary Materials

The following supporting information can be downloaded at: https://www.mdpi.com/article/10.3390/polym15193888/s1. Figure S1: Specimens printed at 2 mm/s. (a) using a needle diameter of 14 G without using silicone oil to reduce the friction forces within the needle when printing. (b) without using any needle and printing directly from the syringe. (c) using a needle diameter of 14 G with silicone oil; Figure S2: EPS-acetone mixture (EPS-ink), where the excess of acetone is observed as a supernatant; Video S1: EPS-ink preparation from the mixture of EPS waste and acetone; Video S2: Video with optimal 3D printing conditions: needle diameter of 14 G, printing speed of 12 mm/s, and bed temperature of 25 °C. In addition, the silicone oil wash was also effective.
