# Supplementary material for: Novel and Accessible Physical Recycling for Expanded Polystyrene Waste with the Use of Acetone as a Solvent and Additive Manufacturing (Direct Ink-Write 3D Printing)
Source: Polymers (Basel). 2023 Sep 26;15(19):3888. doi: 10.3390/polym15193888 (PMC10575267; doi:10.3390/polym15193888)
Supplement: Supplementary file 1 [file polymers-15-03888-s001.zip › polymers-2561356-supplementary.pdf]

Supplementary Information

Novel and Accessible Physical Recycling for Expanded Polystyrene Waste with the Use of Acetone as a Solvent and Additive Manufacturing (Direct Ink-Write 3D Printing)

Rubén García-Sobrino [1,†], Alejandro Cortés [1,*,†], Rocío Calderón-Villajos [1], Jorge G. Díaz [2] and Marta Muñoz [1,*]

1 Department of Applied Mathematics, Materials Science and Engineering and Electronic Technology, Universidad Rey Juan Carlos, Calle Tulipán s/n, 28933 Móstoles, Spain; ruben.sobrino@urjc.es (R.G.-S.)

2 School of Mechanical Engineering, Industrial University of Santander, Bucaramanga 680002, Colombia; jgdiazro@correo.uis.edu.co

* Correspondence: alejandro.cortes@urjc.es (A.C.); marta.munoz@urjc.es (M.M.)

† These authors contributed equally to this work.

KEYWORDS: Circular economy; Plastic waste recycling; Expanded polystyrene; Additive Manufacturing; Direct Ink Write

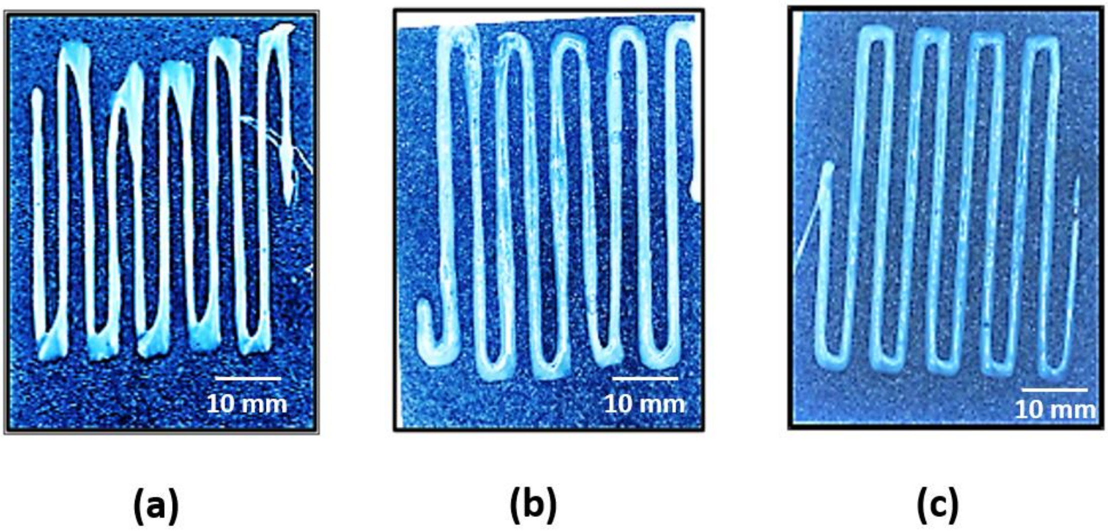

Supplementary Figure S1. Specimens printed at 2 mm/s. **a)** using a needle diameter of 14 G, without using silicone oil for reducing the friction forces within the needle when printing. **b)**

without using any needle and printing directly from the syringe. **c)** using a needle diameter of 14 G with silicone oil.

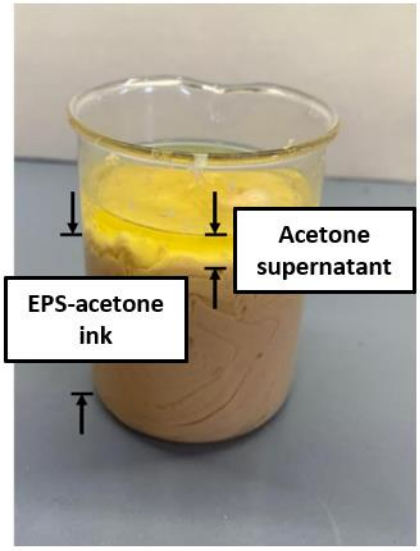

Supplementary Figure S2. EPS-acetone mixture (EPS-ink), where the excess of acetone observed as supernatant.
